# P03-09 Cross-sectional associations between physical activity pattern, sports participation, screen time and mental health in Swedish adolescents

**DOI:** 10.1093/eurpub/ckac095.045

**Published:** 2022-08-29

**Authors:** Karin Kjellenberg, Örjan Ekblom, Johan Ahlen, Björg Helgadóttir, Gisela Nyberg

**Affiliations:** 1 Department of Physical activity and Health, The Swedish School of Sport and Health Sciences, Stockholm, Sweden; 2 Department of Global Public Health, Karolinska Institutet, Stockholm, Sweden; 3 Department of Clinical Neuroscience, Karolinska Institutet, Stockholm, Sweden

**Keywords:** Sedentary Behavior, Screen time, Accelerometry, Mental health

## Abstract

**Background:**

Poor mental health among youth is a public health concern. As half of the mental disorders occur before or during adolescence it is important to investigate how modifiable lifestyle factors are associated with mental health in this population. The association between physical activity patterns and mental health has been studied before but most studies rely on self-reported physical activity. This study aimed to investigate the associations between device-measured physical activity patterns, sports participation, screen time, and mental health in Swedish adolescents.

**Methods:**

Cross-sectional data were collected from 1139 adolescents aged 13-14 in 2019. Data on physical activity patterns were collected using accelerometers for one week. Screen time and sports participation were self-reported by the students. Anxiety and health-related quality of life were assessed using a short version of the Spence Children's Anxiety Scale and Kidscreen-10.

**Results:**

A positive association between time spent in moderate-to-vigorous-physical activity during the whole week and health-related quality of life was found (B = 0.03, CI: 0.01, 0.05 and B = 0.04, CI: 0.02, 0.07), whereas sedentary time during the whole week (B=-0.02, CI: -0.03, -0.01 and B=-0.02, CI: -0.03, -0.01) and high screen time on weekdays (B=-3.50, CI: -4.79, -2.22 and B=-1.54, CI: -2.66 -0.41) were associated with low health-related quality of life in girls and boys respectively. Although the effect sizes generally were small, the largest effect sizes were observed between the high/low MVPA group in boys (Cohen's d -0.51) and high/low screen time group on weekdays in girls (Cohen's d 0.59). With regards to anxiety, high moderate-to-vigorous-physical activity during leisure time on weekdays was associated with low anxiety scores in girls (B=-0.09, CI: -0.13, -0.05) and boys (B=-0.4, CI: -0.07, -0.01). Gender differences were observed, boys participating in organized sports had lower anxiety (B= -1.81 CI: -3.49, -0.13) whereas girls who reported high screen time on weekdays had high anxiety (B = 4.06, CI: 1.94, 6.18).

**Conclusions:**

Our results could create a paradigm for future studies to decide which types of PA patterns and time domains to target in intervention studies with the aim to improve mental health among adolescents.

